# Effects of beta-alanine supplementation on exercise performance and related physiological outcomes in women: a systematic review and meta-analysis

**DOI:** 10.3389/fnut.2026.1857513

**Published:** 2026-06-11

**Authors:** Jinfa Gu, Yaolu Wei, Jie Huang, Yifeng Bu, Shihao Xie

**Affiliations:** 1 School of Physical Education, Jiangsu Normal University, Xuzhou, Jiangsu, China; 2 School of Physical Education, Henan Polytechnic University, Jiaozuo, Henan, China; 3 Department of Pharmacy, Ezhou Central Hospital, Ezhou, Hubei, China; 4 Faculty of Education and Sports Studies, Universiti Malaysia Sabah, Kota Kinabalu, Sabah, Malaysia; 5 School of Physical Education and Health Engineering, Taiyuan University of Technology, Taiyuan, China

**Keywords:** beta-alanine, exercise performance, meta-analysis, time to exhaustion, VO_2_max, women

## Abstract

**Background:**

Beta-alanine is widely used as an ergogenic aid in sports nutrition, yet most evidence comes from male or mixed-sex samples. Its effects in women therefore remain unclear. This systematic review and meta-analysis examined the effects of beta-alanine supplementation on exercise performance and related outcomes in women.

**Methods:**

This review followed PRISMA 2020 guidelines and was registered in PROSPERO (CRD420261362733). PubMed, Web of Science, Scopus, the Cochrane Library, and Embase were searched from inception to April 30, 2026. Randomized controlled studies in women comparing beta-alanine with placebo or control were included. Outcomes included time to exhaustion (TTE), maximal or peak oxygen uptake, peak power, anaerobic performance, and body fat percentage. RoB 2 and GRADE were used to assess risk of bias and certainty of evidence, respectively. Random-effects meta-analyses were conducted using standardized mean differences with 95% confidence intervals.

**Results:**

Twelve reports from 11 independent randomized controlled trials involving 312 women were included. Beta-alanine supplementation showed a pooled effect in favor of TTE (8 studies, *N* = 187; SMD = 0.49, 95% CI 0.20 to 0.79; *p* = 0.001; I^2^ = 0%). For the remaining outcomes, pooled estimates were imprecise, with 95% confidence intervals including trivial and, where applicable, potentially meaningful effects. Moreover, certainty of evidence was very low for all outcomes after downgrading for risk of bias, imprecision, and publication bias; peak power and anaerobic performance were additionally downgraded for indirectness.

**Conclusion:**

Beta-alanine supplementation may improve TTE in trials conducted in women, but current evidence does not support clear pooled effects on peak power, anaerobic performance, VO₂max and VO₂peak, or body fat percentage. Overall, these findings suggest an outcome-specific response pattern, although confidence in the evidence remains limited because of the small evidence base and very low certainty across outcomes.

**Systematic review registration:**

https://www.crd.york.ac.uk/PROSPERO/view/CRD420261362733, identifier PROSPERO (CRD420261362733).

## 1 Introduction

Beta-alanine is one of the most widely used dietary supplements in sports nutrition and has attracted sustained research interest because of its potential ergogenic properties (1, 2). As the rate-limiting precursor in carnosine synthesis, it has the potential to raise intramuscular concentrations of carnosine and increase intracellular buffering capacity (3–5). During high-intensity exercise, this mechanism may help attenuate the adverse effects associated with acid–base disturbance and delay the onset of fatigue (6, 7). Accordingly, beta-alanine is often considered a nutritional strategy with the potential to improve exercise performance (1, 5).

Although research on beta-alanine has expanded substantially, available evidence does not support uniformly favorable effects across all exercise-related outcomes. Previous studies and systematic reviews suggest that potential benefits are more likely to appear in outcomes related to exercise tolerance or exercise capacity than in some performance-based measures, for which the findings remain equivocal (5, 8, 9). This trend indicates that the influence of beta-alanine might not lead to consistent or equal enhancements in all areas of athletic performance (10).

Another important limitation of the existing literature is that most evidence has been derived from male or mixed-sex samples, whereas female-specific results are often underreported or not presented separately (11–14). Consequently, conclusions from the broader beta-alanine literature cannot be assumed to apply directly to women (1, 5, 15). This issue is particularly relevant because the interpretation and practical applicability of supplementation effects in women may be influenced by female-specific physiological and methodological factors that are often insufficiently reported in primary studies (16, 17). To date, direct extrapolation from predominantly male or mixed-sex evidence to women may be inappropriate or, at a minimum, insufficiently supported (5).

An independent synthesis of evidence in women is also needed. The reason is that women who participate in exercise and sport represent a broad range of populations, including sedentary or untrained individuals, recreationally active participants, collegiate athletes, and highly trained or elite athletes (18–21). Currently, even though the outcomes examined in beta-alanine research are multidimensional and may include exercise tolerance, aerobic capacity, anaerobic performance, peak power, and body composition, the strength and consistency of evidence appear to differ across these domains (8, 10). Accordingly, the present review focuses on five prespecified outcomes: time to exhaustion (TTE), maximal or peak oxygen uptake (VO₂max and VO₂peak), peak power, anaerobic performance, and body fat percentage (12).

As far as the present body of literature, no synthesis with quantitative pooling has been identified that is limited to randomized controlled studies and the establishment of the effects of beta-alanine on the mentioned outcomes in female populations. This evidence gap warrants attention because a women-focused synthesis may provide a more appropriate basis for interpretation compared to relying on conclusions drawn primarily from male or mixed-sex samples. Furthermore, instead of assuming a broadly consistent ergogenic effect, the current synthesis may help clarify whether the current women-specific evidence is more consistent with benefits in selected outcome domains than with a uniform response across exercise-related outcomes (13).

Therefore, the present systematic review and meta-analysis aims to evaluate the effects of beta-alanine supplementation on exercise performance and related outcomes in women. Specifically, randomized controlled trials (RCTs) were synthesized for TTE, VO₂max and VO₂peak, peak power, anaerobic performance, and body fat percentage. Given the inconsistency of outcome-specific responses reported in the existing literature, the present review did not presuppose a uniform ergogenic effect of beta-alanine in women. Instead, it examined whether the available evidence is more consistent with an outcome-specific pattern of response. By establishing a more women-focused evidence framework, this review seeks to provide a more rigorous and appropriately qualified basis for the interpretation and practical application of beta-alanine supplementation in women.

## 2 Materials and methods

### 2.1 Registration and reporting standards

In line with the PRISMA 2020 reporting guidelines and using the Cochrane Handbook of Systematic Reviews of Interventions to guide the methodological conduct and documentation of this investigation, the implementation and reporting of this study adhered to the generally accepted standards of rigor and transparency (22). A study protocol was registered in the International Prospective Register of Systematic Reviews (PROSPERO), in which the protocol is indexed using the reference number CRD420261362733.

### 2.2 Search strategy

Two reviewers independently carried out a comprehensive and methodically structured search of the existing academic literature in PubMed, Web of Science, Scopus, the Cochrane Library, and Embase from inception to 30 April 2026. The retrieval approach incorporated both standardized indexing terminology, such as MeSH and Emtree, together with free-text keywords related to beta-alanine, women, exercise performance, and the prespecified outcomes of interest. Search terms were combined using Boolean operators as appropriate. Moreover, all included articles and the corresponding reviews were manually searched through their reference lists to determine any other eligible studies. Only articles in English were considered, and no limitation with regard to the year of publication was applied. The complete database-specific search strategies, including exact syntax, field tags, search dates, filters, and limits, are provided in Supplementary Table S7.

### 2.3 Study selection and screening process

After importing all electronically retrieved records into a reference management software and subsequently eliminating the records containing duplicates, study selection was done in two separate phases according to set eligibility criteria. Two reviewers then independently screened the titles and abstracts, and full-text articles of potentially eligible studies were screened for inclusion. Any disagreement was resolved through discussion with the assistance of a third reviewer in case no consensus was achieved.

### 2.4 Eligibility criteria

#### 2.4.1 Inclusion criteria

Participants: Female participants, or studies that permit the separate extraction of data specific to women.

Intervention: Repeated beta-alanine supplementation, including multi-day or multi-week protocols, as the core intervention of interest. Studies involving co-interventions were eligible only when the independent effect of beta-alanine could be isolated.

Comparators: Placebo, control, or a comparator allowing evaluation of the independent effect of beta-alanine.

Outcomes: At least one prespecified outcome of interest, including TTE, VO₂max and VO₂peak, peak power, anaerobic performance (power- and time-based measures), or body fat percentage.

Study design: Eligibility was restricted exclusively to RCTs.

#### 2.4.2 Exclusion criteria

Exclusion criteria were applied to studies that enrolled only male participants, included mixed-sex samples without separately extractable female data, did not evaluate beta-alanine as the core intervention, used multi-ingredient supplementation protocols from which the independent effect of beta-alanine could not be isolated, lacked an eligible comparator, or did not report extractable data for at least one prespecified outcome. Animal studies, review articles, conference abstracts, and studies without sufficient data for quantitative synthesis were also excluded. Studies using acute single-dose beta-alanine ingestion or acute-only supplementation protocols were excluded.

### 2.5 Data extraction and data handling

Using a predesigned standardized form, data extraction was carried out independently by two reviewers. The publication year, sample size, study design, characteristics of participants, training status, exercise modality, and information on intervention and comparator conditions were considered, including beta-alanine dose, supplementation duration, and any relevant co-interventions. Data for the prespecified outcomes were also extracted.

Outcomes were categorized as TTE, VO₂max and VO₂peak, peak power, anaerobic performance, and body fat percentage. VO₂max and VO₂peak were analyzed within the same outcome domain as measures of maximal or peak oxygen uptake, while the combined terminology was retained to reflect methodological differences across studies. Anaerobic performance was defined as power- and time-based measures derived from short-duration, high-intensity exercise tasks.

In any conflicts that arose in the data extraction, the two reviewers resolved the differences by discussing the contentious issues with each other and, in cases of need, resorted to a third reviewer. For continuous outcomes, post-intervention means and standard deviations were preferentially extracted because these data were most consistently available across studies. When change scores or adjusted estimates were the only extractable data, they were used only when a comparable effect estimate could be derived. Standard errors were converted to standard deviations using the corresponding group sample size where necessary. No within-participant correlations were imputed for change-score calculations, and no crossover trials requiring paired-effect estimation were included. To avoid double-counting participants within any single pooled analysis, only one effect size per study was included for each outcome domain. For studies with co-interventions or multi-arm designs, only the beta-alanine-only versus matched placebo/control comparison under the same background condition was extracted. Combination arms involving beta-alanine plus another active supplement, such as sodium bicarbonate or creatine, and other non-relevant arms were excluded, and no shared comparator group was used more than once within the same pooled outcome. When multiple measures were reported within the same outcome domain, we selected the measure that best matched the prespecified domain definition and was least likely to overlap conceptually with other included outcomes. Detailed arm-level extraction decisions for multi-arm and co-intervention studies are provided in Supplementary Table S4.

### 2.6 Risk of bias and methodological quality assessment

The assessment of the risk of bias was conducted using the Cochrane Risk of Bias 2 (RoB 2) tool (23), which comprised five domains: the randomization process, deviations from intended interventions, the completeness of outcome data, outcome measurement, and selection of the reported result. The independent evaluations were done by two reviewers, and disagreements between the two were resolved by discussion and, in cases where it was deemed necessary, involved a third reviewer. RoB 2 application was carried out at the outcome level, and general bias risk judgments were made using the RoB 2 algorithm as low risk, some concerns, or high risk. Outcome-measurement judgments considered blinding success, reported side effects, matching of the placebo to the active supplement, supplement form, and assessor blinding, where available. When these details were insufficient to rule out expectancy-related influences on performance outcomes, this domain was judged as raising some concerns.

### 2.7 Certainty of evidence (GRADE)

The Grading of Recommendations Assessment, Development and Evaluation (GRADE) framework was used to determine the certainty of evidence for each outcome separately (24). Because all included studies were randomized controlled trials, the certainty of evidence initially started as high. Certainty was downgraded when appropriate for risk of bias, inconsistency, indirectness, imprecision, and publication bias. Because fewer than 10 studies contributed to each pooled outcome, publication bias could not be formally assessed and was therefore judged qualitatively within the GRADE framework. Publication bias was downgraded when it could not be adequately ruled out. Two reviewers independently performed the assessment, and disagreements were resolved through discussion and consensus. An outcome-specific GRADE Summary of Findings table was prepared to present pooled estimates and the rationale for downgrading decisions across the five GRADE domains.

### 2.8 Statistical analysis

Random-effects models were used to conduct all meta-analyses. Continuous outcomes were pooled as standardized mean differences (SMDs) with 95% confidence intervals, using Hedges’ small-sample correction. The between-study variance was estimated using the DerSimonian–Laird method. Between-study heterogeneity was summarized using τ^2^ and I^2^. Prediction intervals were generated for forest plots and interpreted cautiously. For anaerobic performance outcomes based on time, effect directions were aligned before pooling to ensure consistent interpretation. Statistical heterogeneity was evaluated based on the I^2^ statistic, which has thresholds of approximately 25, 50, and 75% that represent low, moderate, and high heterogeneity, respectively. A leave-one-out approach was used to conduct sensitivity analyses. Additional sensitivity analyses compared the results from random-effects and fixed-effect models. REML-based random-effects sensitivity analyses were also conducted to assess the robustness of the primary DerSimonian–Laird estimates. Prespecified subgroup analyses were conducted for TTE according to training status, beta-alanine dose, and TTE exercise-test modality. Subgroup analyses interpretation was viewed as exploratory and not confirmatory because of the small number of studies in each subgroup. Because fewer than 10 studies were available for each pooled analysis, funnel plots and statistical tests for small-study effects were not performed. This limitation was considered qualitatively in the GRADE assessment rather than being interpreted as evidence that publication bias was absent. In addition, statistical significance was defined by a two-sided *p*-value of < 0.05. Analyses were conducted using R software (version 4.5.2) with the meta package.

## 3 Results

### 3.1 Study selection

**Figure 1:**
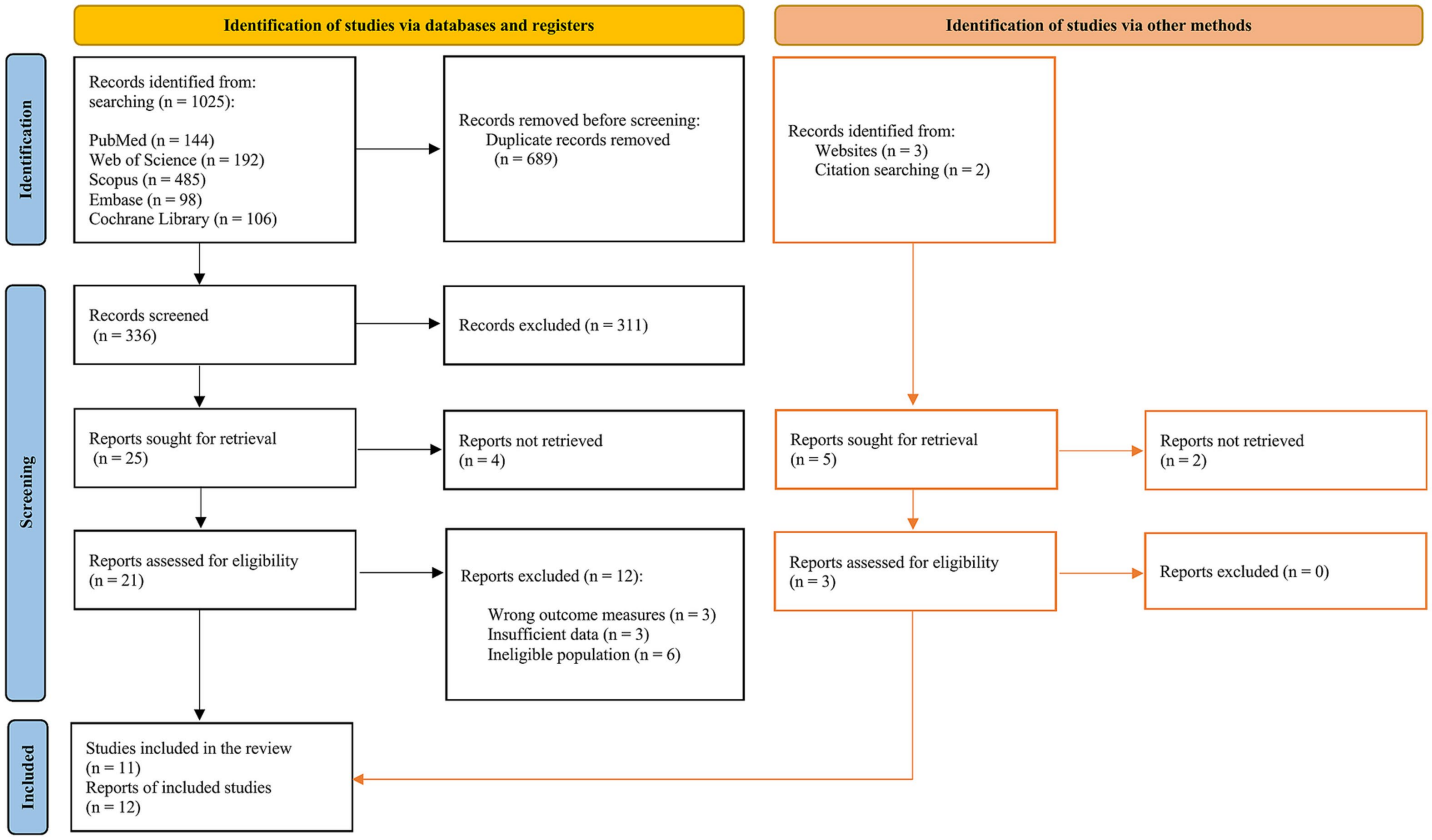
Preferred reporting items for systematic reviews flow diagram. Two reports by Glenn et al. (27, 28) originated from the same randomized controlled trial and were therefore treated as a single study in the analysis.

Database searching identified 1,025 records, and an additional 5 records were identified through other methods (Figure 1). After removing 689 duplicate records from the database search results, 336 records remained for screening. Of these, 311 records were excluded, and 25 reports were sought for retrieval. Four reports from database searching were not retrieved, leaving 21 reports for eligibility assessment. In addition, 5 reports identified through other methods were sought for retrieval, of which 2 were not retrieved, leaving 3 reports for eligibility assessment. After full-text review, 12 reports were excluded for the following reasons: wrong outcome measures (*n* = 3), insufficient data (*n* = 3), and ineligible population (*n* = 6). Finally, 12 reports from 11 independent RCTs were included in the review. A report-to-study mapping table, including companion reports and participant-overlap decisions, is provided in Supplementary Table S5.

### 3.2 Study characteristics

The review included 12 reports from 11 independent RCTs, comprising 312 female participants in total (18–21, 25–32). Across the included comparisons used in the meta-analysis, comparison-specific sample sizes ranged from 15 to 34 participants, and reported participant age values ranged from 18 to 54 years. Participant characteristics varied markedly across studies, spanning sedentary and untrained women, recreationally active individuals, collegiate athletes, and highly trained or elite female athletes. Exercise modalities were also diverse, including basketball, soccer, cycling, resistance training, mixed training settings, and non-athletic populations.

Considerable variation was observed in the beta-alanine supplementation protocols. Prescribed doses generally fell between 1.6 and 6.4 g/day, although some studies used divided daily doses or protocols scheduled around training sessions. Supplementation durations ranged from 21 days to 8 weeks; however, the limited number of studies precluded a reliable duration-based analysis.

The outcomes most commonly evaluated among the included studies were TTE, peak power, anaerobic performance (power- and time-based measures), VO₂max and VO₂peak, and body fat percentage. Taken together, these outcomes captured both body-composition responses and exercise performance to beta-alanine supplementation in women with differing training status. Co-interventions varied across studies and included standardized diet or testing procedures, structured training programs, usual-activity maintenance, or no additional training intervention; menstrual/contraceptive status was not reported, and blinding or side-effect information was limited or inconsistently reported (Table 1).

**Table 1:** Characteristics of the included randomized controlled trials and comparisons used in the meta-analysis. (A) summarizes study and participant characteristics. (B) summarizes intervention, comparator, co-intervention, reporting, extracted outcome, and meta-analysis comparison details.

(A) Study and participant characteristics					
Study	Study design	Sample size (BA/comparator)	Age (years)	Training status	Exercise modality
Adamczewski et al. (25)	RCT	17/17	21.4 ± 4.2	Highly trained or elite female basketball players	Basketball
Walter et al. (32)	RCT	14/19	21.8 ± 3.7	Recreationally active women	Cycling
Gholami et al. (26)	RCT	11/11	21.7 ± 1.2	Collegiate female basketball players	Basketball
Ribeiro et al. (21)	RCT	12/12	18 ± 1	Elite international U20 female footballers	Soccer
Hooshmand et al. (18)	RCT	17/17	20–45	Sedentary overweight women	Non-athletic population
Rosas et al. (29)	RCT	8/8	23.7 ± 2.4	Amateur female soccer players	Soccer
Outlaw et al. (20)	RCT	7/8	21.0 ± 2.2	Untrained collegiate females	Resistance training
Glenn et al. (27) (masters cyclists trial)	RCT; companion reports from the same trial	11/11	54 ± 2/53 ± 1	Female masters cyclists	Cycling
Kresta et al. (19)	RCT; multi-arm trial	8/7	21.5 ± 2.8	Recreationally active females	Mixed training background
Smith et al. (30)	RCT	13/11	21.7 ± 2.1	Moderately trained women	Mixed training background
Stout et al. (31)	RCT	11/11	27.4 ± 6.1	Women	Unspecified

(B) Intervention, comparator, and extracted outcome details.								
Study	Supplementation protocol (dose/duration)	Comparator	Supplement form	Co-intervention/background condition	Menstrual/contraceptive status	Blinding/side effects	Outcome measures	Specific comparison used in meta-analysis
Adamczewski et al. (25)	6.4 g/day; 28 days	Placebo	BA vs. placebo capsules	Standardized diet before testing	NR	NR	TTE; Peak Power; Anaerobic Performance	BA+PLSB vs. PLBA+PLSB; SB-containing arms excluded
Walter et al. (32)	1.5 g per dose, 4 doses/day for 21 days, then 2 doses/day for 21 days	Placebo / control	BA + dextrose powder	6 weeks HIIT cycling	NR	NR	VO₂max; Body Fat Percentage	BA vs. matched placebo/control under the same HIIT background condition
Gholami et al. (26)	6.4 g/day; 28 days	Isocaloric placebo	BA vs. placebo (dextrose)	Exhaustive exercise testing	NR	NR	TTE; Peak Power; VO₂max; Body Fat Percentage	BA vs. isocaloric placebo
Ribeiro et al. (21)	6.4 g/day; 21 days	Maltodextrin placebo	Sustained-release BA	3-week standardized football-specific training camp	NR	NR	Anaerobic Performance	BA vs. maltodextrin placebo under the same training-camp condition
Hooshmand et al. (18)	1.6 g/day; 42 days	Placebo	BA supplement	Usual diet and daily activity maintained	NR	NR	TTE; Body Fat Percentage	BA vs. placebo
Rosas et al. (29)	4.8 g/day; 6 weeks	Placebo	Oral BA supplementation	Plyometric training	NR	Blinding success / side effects NR	Anaerobic Performance	BA vs. placebo under the same plyometric-training condition
Outlaw et al. (20)	3.4 g before training sessions; 8 weeks	Placebo	BA before training sessions	8 weeks of resistance training	NR	Blinding success / side effects NR	TTE; Peak Power; VO₂max; Body Fat Percentage	BA vs. placebo under the same resistance-training condition
Glenn et al. (27) (masters cyclists trial)	3.2 g/day; 28 days	Placebo	BA + dextrose	No co-intervention; usual training maintained	NR	Blinding efficacy tested; paresthesia reported in one participant	TTE; VO₂peak; Body Fat Percentage	BA vs. placebo; overlapping outcomes extracted only once
Kresta et al. (19)	6.1 ± 0.7 g/day; 28 days	Placebo	BA sustained-release capsules	4-arm trial; no additional training intervention	NR	NR	TTE; Peak Power; VO₂max; Anaerobic Performance; Body Fat Percentage	BA-only arm vs. matched placebo; non-isolated active-supplement arms excluded
Smith et al. (30)	4.8 g/day; 28 days	Placebo	CarnoSyn tablets	40-min treadmill run to induce oxidative stress	NR	NR	TTE	BA vs. placebo
Stout et al. (31)	3.2 g/day for 7 days, then 6.4 g/day for 21 days; 28 days total	Placebo	CarnoSyn	No co-intervention	NR	NR	TTE	BA vs. placebo

For multi-arm trials, only comparisons relevant to isolated beta-alanine supplementation were used in the pooled analyses. Glenn et al. (27) refers to two companion reports from the same 28-day beta-alanine supplementation trial in female masters cyclists; overlapping outcomes were extracted only once. For the Glenn companion reports, the sample size used in the meta-analysis was 11/10 for TTE and VO₂peak because one placebo-group participant withdrew due to repeated headaches and was not included in the final analyses; body fat percentage used 11/11.

BA, beta-alanine; NR, not reported; PLBA, placebo beta-alanine; PLSB, placebo sodium bicarbonate; SB, sodium bicarbonate; TTE, time to exhaustion; VO₂max, maximal oxygen uptake; VO₂peak, peak oxygen uptake.

### 3.3 Risk of bias results

**Figure 2:**
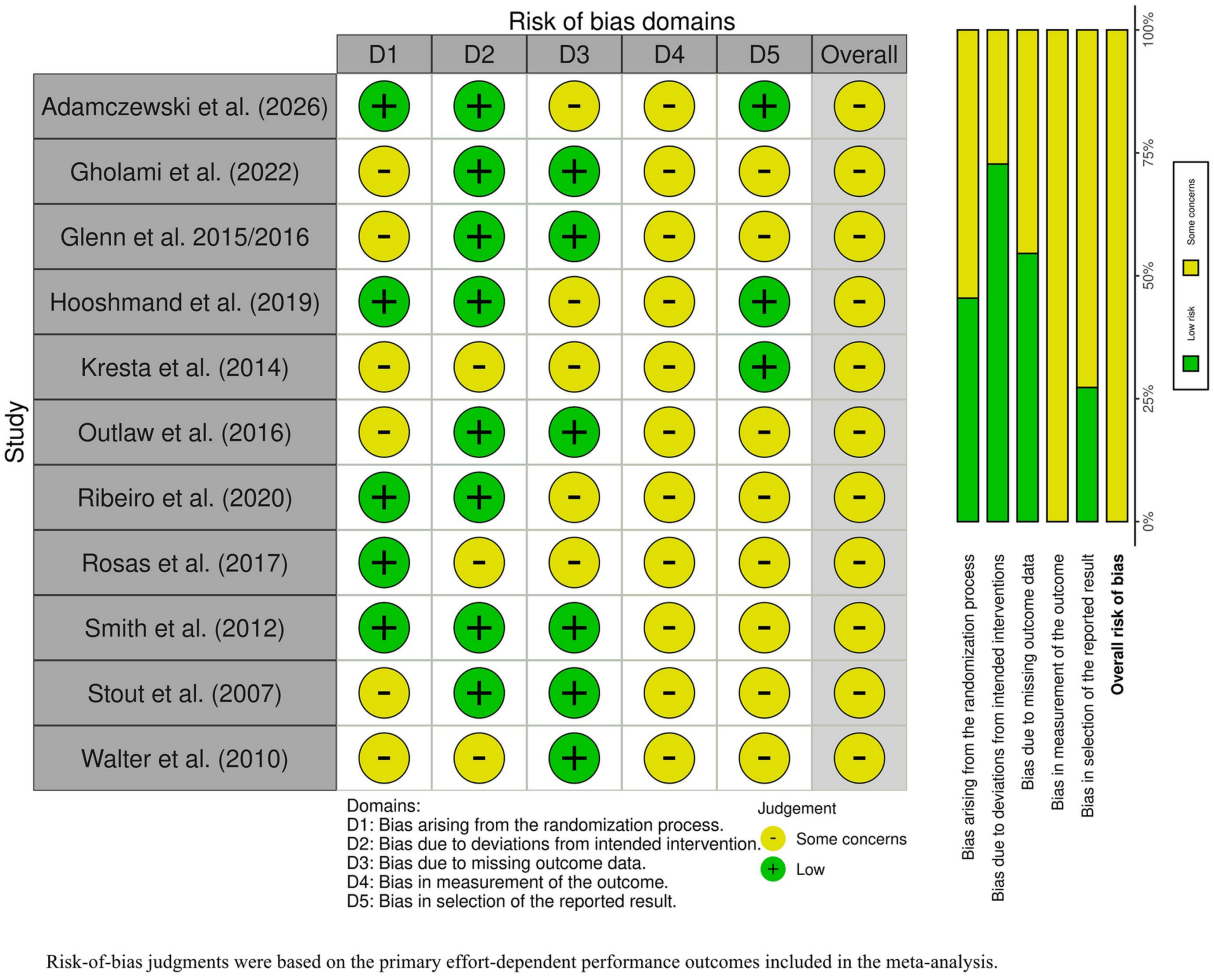
Risk-of-bias evaluation of included RCTs based on the Cochrane risk of bias 2 tool. D1, bias arising from the randomization process; D2, bias due to deviations from intended interventions; D3, bias due to missing outcome data; D4, bias in measurement of the outcome; D5, bias in selection of the reported result.

Using the Cochrane Risk of Bias 2 tool, all included studies were judged as raising some concerns overall, with no study rated as low risk or high risk overall (Figure 2). Domain-level concerns were most frequent for outcome measurement (D4, 100%), reflecting limited reporting of blinding success, side effects, matching of the placebo to the active supplement, supplement form, and assessor blinding. Concerns were also identified for selection of the reported result (D5, 72.7%), the randomization process (D1, 54.5%), and missing outcome data (D3, 45.5%). In contrast, most studies were judged as low risk for deviations from intended interventions (D2, 72.7%). Full outcome-level RoB 2 judgments and blinding-related information are provided in Supplementary Table S6.

### 3.4 Overall effects

**Figure 3:**
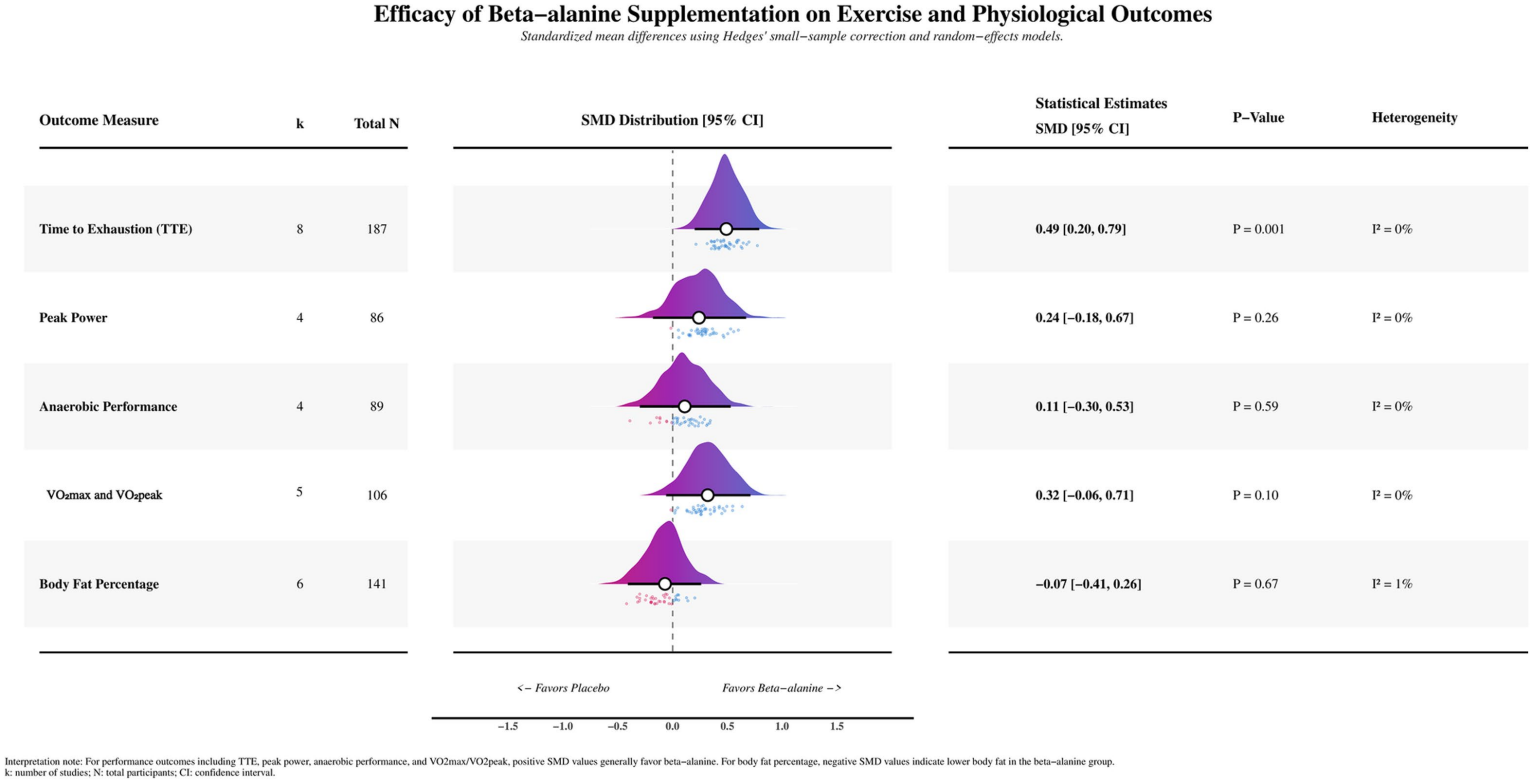
Summary plot of pooled estimates for the effects of beta-alanine supplementation on TTE, peak power, anaerobic performance, VO_2_max and VO_2_peak, and body fat percentage.

Exercise performance outcomes showed a pooled effect favoring beta-alanine supplementation for TTE (8 studies, *N* = 187; SMD = 0.49, 95% CI 0.20 to 0.79; *p* = 0.001; I^2^ = 0%). For peak power (4 studies, *N* = 86; SMD = 0.24, 95% CI −0.18 to 0.67; *p* = 0.26; I^2^ = 0%) and anaerobic performance (4 studies, *N* = 89; SMD = 0.11, 95% CI −0.30 to 0.53; *p* = 0.59; I^2^ = 0%), the confidence intervals crossed the null and included both trivial and potentially meaningful effects. A similar pattern was observed for VO₂max and VO₂peak (5 studies, *N* = 106; SMD = 0.32, 95% CI −0.06 to 0.71; *p* = 0.10; I^2^ = 0%) and body fat percentage (6 studies, *N* = 141; SMD = −0.07, 95% CI −0.41 to 0.26; *p* = 0.67; I^2^ = 1%). Figure 3 summarizes the pooled estimates across outcomes. Detailed forest plots with study-level estimates and prediction intervals for all outcomes are provided in Supplementary Figure S1.

### 3.5 Subgroup analyses for TTE

**Figure 4:**
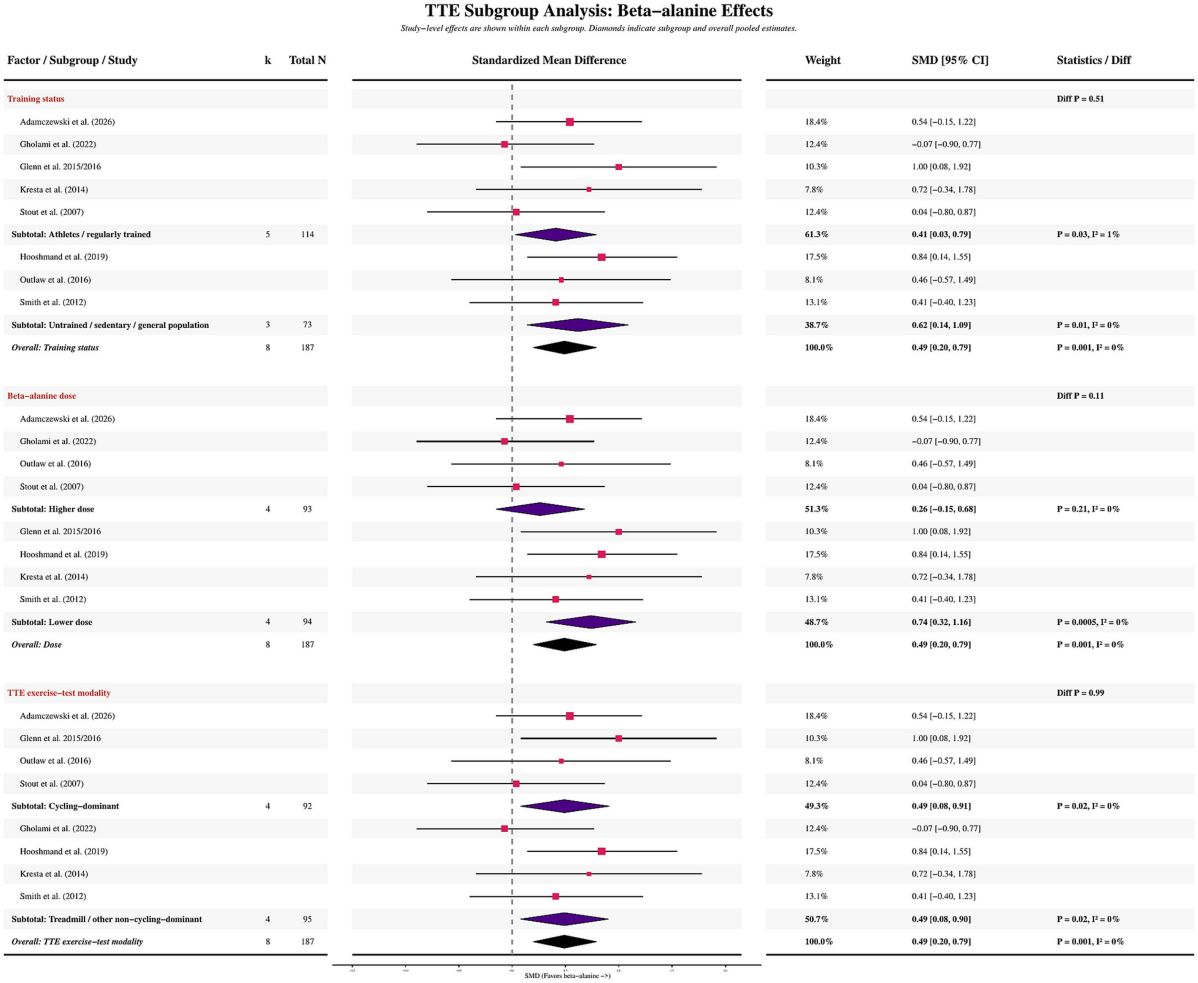
Subgroup analyses of the effects of beta-alanine supplementation on TTE according to training status, beta-alanine dose, and TTE exercise-test modality.

Subgroup analyses for TTE were conducted according to training status, beta-alanine dose, and TTE exercise-test modality. Although some subgroups showed statistically significant pooled effects, no statistically meaningful differences were detected between subgroups (all interaction *p* > 0.05). Therefore, these subgroup findings should be interpreted as exploratory and hypothesis-generating only, and no conclusion can be drawn regarding differential effects across dose, training status, or TTE exercise-test modality. Specifically, the apparent effect in the lower-dose subgroup does not indicate that lower doses are more effective, as formal between-subgroup interactions did not support this interpretation. Subgroup analyses by training status, dose, and TTE exercise-test modality are presented in Figure 4.

### 3.6 Sensitivity analysis

The leave-one-out analyses, fixed-effect versus random-effects model comparisons, and REML-based random-effects sensitivity analyses did not materially alter the overall findings. No individual study materially changed the direction, statistical significance, or overall magnitude of the pooled effects. Detailed numerical results are provided in Supplementary Tables S1–S3.

### 3.7 Certainty of evidence (GRADE)

Table 2 summarizes the certainty of evidence for each primary outcome using the GRADE framework. Certainty was rated as very low for all outcomes. All outcomes were downgraded for risk of bias because the contributing randomized controlled trials were judged as having some concerns in the overall RoB 2 assessment. All outcomes were also downgraded for imprecision because of the limited total sample size and/or wide confidence intervals around the pooled estimates. Publication bias was downgraded because publication bias and small-study effects could not be adequately ruled out, given the small number of studies contributing to each outcome and the limited evidence base. Peak power and anaerobic performance were additionally downgraded for indirectness because the pooled outcomes combined different exercise protocols, testing methods, or outcome definitions. Inconsistency was not downgraded because statistical heterogeneity was minimal across pooled outcomes.

**Table 2:** GRADE summary of findings for primary outcomes.

Outcomes	No. of studies	No. of participants	Pooled estimate, SMD [95% CI]	Risk of bias	Inconsistency	Indirectness	Imprecision	Publication bias	Certainty (GRADE)
TTE	8 RCTs	187	0.49 [0.20, 0.79]	Serious^a^	Not serious^b^	Not serious	Serious^d^	Seriousᵉ	⊕◯◯◯ Very low
Body fat percentage	6 RCTs	141	−0.07 [−0.41, 0.26]	Serious^a^	Not serious^b^	Not serious	Serious^d^	Seriousᵉ	⊕◯◯◯ Very low
Peak power	4 RCTs	86	0.24 [−0.18, 0.67]	Serious^a^	Not serious^b^	Serious^c^	Serious^d^	Seriousᵉ	⊕◯◯◯ Very low
Anaerobic performance	4 RCTs	89	0.11 [−0.30, 0.53]	Serious^a^	Not serious^b^	Serious^c^	Serious^d^	Seriousᵉ	⊕◯◯◯ Very low
VO₂max and VO₂peak	5 RCTs	106	0.32 [−0.06, 0.71]	Serious^a^	Not serious^b^	Not serious	Serious^d^	Seriousᵉ	⊕◯◯◯ Very low

GRADE, Grading of Recommendations Assessment, Development and Evaluation; RCT, randomized controlled trial; SMD, standardized mean difference; CI, confidence interval; TTE, time to exhaustion. Randomized controlled trials started as high-certainty evidence. Certainty was assessed separately for each outcome and was not downgraded below very low.

a. Downgraded one level for risk of bias because the contributing randomized controlled trials were judged as having some concerns in the overall RoB 2 assessment. b. Not downgraded for inconsistency because statistical heterogeneity was minimal across pooled outcomes. c. Downgraded one level for indirectness because the pooled outcome combined different exercise protocols, testing methods, or outcome definitions. d. Downgraded one level for imprecision because of the limited total sample size and/or wide confidence interval around the pooled estimate. e. Downgraded one level for publication bias because publication bias and small-study effects could not be adequately ruled out. Fewer than 10 studies contributed to each pooled outcome, which precluded reliable formal assessment of small-study effects, and the limited evidence base increased uncertainty regarding selective publication or unavailable study results.

## 4 Discussion

### 4.1 Main findings

The impact of beta-alanine supplementation on exercise performance and the outcomes associated with it in women was investigated using a systematic review and a quantitative approach of meta-analysis. The pooled effect estimate for TTE was in favor of beta-alanine supplementation, whereas no statistically meaningful pooled effects were identified for peak power, anaerobic performance, VO₂max and VO₂peak, or body fat percentage. Sensitivity analyses indicated that the main findings were generally stable. However, the certainty of evidence was very low across outcomes. Accordingly, the present findings are better interpreted as a synthesis of the currently available evidence in women than as a basis for strong or definitive conclusions. Despite low levels of statistical heterogeneity, variations were still present among the included studies in participant characteristics, exercise modality, supplementation protocols, and outcome assessment. These findings should therefore be interpreted as evidence from trials conducted in women, rather than as evidence explaining female-specific physiology.

### 4.2 Effects of beta-alanine on TTE in women and possible explanations

The pooled effect estimate for TTE in women was in favor of beta-alanine supplementation. This finding appears broadly consistent with the direction of the previous literature, although most supporting evidence has been derived from mixed-sex or predominantly male samples rather than from women-only studies (9). The present finding should therefore be interpreted as supportive evidence from trials conducted in women, rather than as evidence that women respond differently from men.

One possible explanation is that beta-alanine, as the rate-limiting precursor for carnosine synthesis, can enhance intramuscular carnosine content and thereby enhance intracellular buffering capacity, which may delay fatigue during high-intensity exercise (5). Compared with measures that primarily reflect brief or instantaneous performance output, TTE requires participants to continue exercising until exhaustion and may therefore be more sensitive to disturbances in internal homeostasis. In this context, the potential effects of beta-alanine may be more likely to emerge in outcomes related to exercise tolerance than in performance-based measures (9). However, TTE is a laboratory-derived tolerance-related outcome and should not be interpreted as directly equivalent to sport-specific or competition performance.

This interpretation should nevertheless remain cautious. Previous studies only support a general tendency for capacity- or tolerance-related outcomes to be more responsive, whereas the present review provides supplementary evidence focused specifically on women. Due to very low certainty in the evidence for TTE and the few studies included, a more conservative interpretation is that trials conducted in women suggest that beta-alanine supplementation may show potential benefits in outcomes such as TTE that reflect tolerance to sustained high-intensity exercise.

### 4.3 Interpretation of the absence of significant pooled effects for peak power, anaerobic performance, and VO₂max and VO₂peak

There were no significant pooled effects on peak power or anaerobic performance (8). This observation is largely congruent with other reviews and indicates that the effects of beta-alanine might not be observed across all axes of exercise performance.

One possible explanation is that these outcomes depend more strongly on instantaneous neuromuscular output, phosphagen-based energy provision, and task-specific performance characteristics than on acid–base disturbance or intracellular buffering capacity. Therefore, even if beta-alanine supplementation enhances buffering capacity through increased muscle carnosine, this physiological change may not translate consistently into improvements in brief, explosive, or peak-oriented tasks. Accordingly, the absence of significant pooled effects for peak power and anaerobic performance should not be interpreted as evidence of no effect, but rather as a lack of consistent support in the currently available evidence from trials conducted in women. The corresponding confidence intervals included trivial effects as well as potentially meaningful effects, indicating that the available evidence remains imprecise rather than definitively excluding a possible effect.

It should also be noted that the definition and assessment of anaerobic performance varied across studies and included both power-based and time-based tasks, which may not reflect the same underlying physiological demands. Although effect directions were aligned before pooling, this construct-level heterogeneity, together with broader clinical and methodological differences, may have reduced the ability of the analysis to detect consistent effects.

Similarly, no significant pooled effect was observed for VO₂max and VO₂peak, which appears consistent with the broader literature (8). From a physiological perspective, this result may be plausible because the primary role of beta-alanine appears to relate more closely to intracellular buffering and fatigue attenuation, whereas VO₂max and VO₂peak are more strongly influenced by the upper limits of oxygen transport and utilization. A cautious interpretation is that the available evidence does not currently support a clear effect of beta-alanine supplementation on VO₂max and VO₂peak in trials conducted in women.

### 4.4 Absence of a significant pooled effect on body fat percentage and its implications

The present review did not observe a significant pooled effect of beta-alanine supplementation on body fat percentage in women. Compared with performance-related outcomes, this finding appears to be more consistent with the existing literature. Based on its known functional role, beta-alanine is more likely to influence metabolic buffering and fatigue regulation during exercise than to act directly on body composition. Therefore, the absence of a significant effect on body fat percentage is not unexpected.

Accordingly, the lack of a significant change in body fat percentage is broadly consistent with the direction of the current evidence base (12). A more appropriate interpretation is that, from an applied perspective, this finding helps define the practical boundary of beta-alanine use in women. However, body-composition outcomes may also be influenced by diet, training mode, energy availability, and hormonal status; therefore, the possibility of differential responses under specific training and nutritional conditions cannot be excluded.

At present, even though beta-alanine may have potential value for some exercise tolerance-related outcomes, the available evidence does not indicate a clear effect on body fat percentage.

### 4.5 Significance and implications of the present review

The significance of the present review lies primarily in its focus on trials conducted in women, a population that has been underrepresented in the broader beta-alanine literature. Most previous beta-alanine reviews have been based mainly on male or mixed-sex samples, with women accounting for only a relatively small proportion of participants (13). By synthesizing evidence from studies that enrolled female participants, the present review provides a more directly relevant summary for women than evidence derived predominantly from male or mixed-sex samples.

More broadly, the available evidence from trials conducted in women appears more consistent with an outcome-specific pattern of response than with a uniform ergogenic effect. These implications should be interpreted cautiously given the methodological limitations of the included trials.

### 4.6 Limitations and future directions

There are a number of limitations to be taken into consideration. To begin with, the total number of articles selected was small, and only a small number of studies contributed to each pooled outcome, which reduced precision and contributed to very low certainty of evidence across outcomes. Second, low statistical heterogeneity does not necessarily indicate full comparability across studies, as clinical and methodological differences remained in training background, exercise modality, supplementation dose and duration, and outcome assessment. Third, important female-specific and contextual factors, including menstrual cycle phase, hormonal contraceptive use, dietary details, and specific training periods, were insufficiently reported or controlled in most primary studies. This limits mechanistic interpretation of female-specific physiological responses. In addition, formal statistical assessment of publication bias and small-study effects was not possible because fewer than 10 studies contributed to each pooled outcome. Therefore, publication bias was judged qualitatively and downgraded in the GRADE assessment because it could not be adequately ruled out. Together, these limitations warrant cautious interpretation of the findings.

Further studies are needed with sufficiently powered RCTs that are specific to women, with greater standardization of outcome measures and better reporting of female-specific variables, to clarify the conditions under which beta-alanine supplementation may or may not be beneficial.

## 5 Conclusion

Beta-alanine supplementation may improve TTE in trials conducted in women, whereas current evidence derived from studies performed in female participants does not support clear pooled effects on peak power, anaerobic performance, VO₂max and VO₂peak, or body fat percentage. Overall, the available evidence from trials conducted in women appears more consistent with an outcome-specific pattern of response than with a uniform ergogenic effect. These results should be viewed with caution because of the small number of included studies, very low certainty of evidence, and limited reporting of female-specific and contextual factors. Therefore, the findings should not be interpreted as evidence explaining female-specific physiology. In future research, additional adequately powered RCTs specifically designed for women are needed.

## Data Availability

The original contributions presented in the study are included in the article/supplementary material, further inquiries can be directed to the corresponding author.
