# Supplementary material for: Effects of beta-alanine supplementation on exercise performance and related physiological outcomes in women: a systematic review and meta-analysis
Source: Front Nutr. 2026 Jun 11;13:1857513. doi: 10.3389/fnut.2026.1857513 (PMC13294097; doi:10.3389/fnut.2026.1857513)
Supplement: Supplementary file 1 [file Supplementary_file_1.docx]

**Supplementary Materials**

Main manuscript tables included at the beginning of this combined file: Table 1 and Table 2.

- Supplementary Tables S1–S3. Sensitivity analysis tables.
- Supplementary Table S4. Arm-level extraction decisions for multi-arm and co-intervention studies.
- Supplementary Table S5. Report-to-study mapping table.
- Supplementary Table S6. Outcome-level RoB 2 assessment and blinding-related information.
- Supplementary Table S7. Database-specific search strategies.
- Supplementary Figure S1. Outcome-specific forest plots for exercise and physiological outcomes.

**Supplementary Figure S1 description:** Forest plots showing the standardized mean differences (Hedges’ g) and 95% confidence intervals for five outcomes: time to exhaustion (TTE), peak power, VO₂max and VO₂peak, anaerobic performance, and body fat percentage. Each plot displays individual study-level effects, pooled estimates, heterogeneity statistics, study weights, and prediction intervals.

# Main manuscript Table 1. Characteristics of included RCTs

**Table 1. Characteristics of the included randomized controlled trials and comparisons used in the meta-analysis**

Panel A summarizes study and participant characteristics. Panel B summarizes intervention, comparator, co-intervention, reporting, extracted outcome, and meta-analysis comparison details.

**Panel A. Study and participant characteristics**

| **Study** | **Study design** | **Sample size (BA / comparator)** | **Age (years)** | **Training status** | **Exercise modality** |
| --- | --- | --- | --- | --- | --- |
| Adamczewski et al. (2026) | RCT | 17 / 17 | 21.4 ± 4.2 | Highly trained or elite female basketball players | Basketball |
| Walter et al. (2010) | RCT | 14 / 19 | 21.8 ± 3.7 | Recreationally active women | Cycling |
| Gholami et al. (2022) | RCT | 11 / 11 | 21.7 ± 1.2 | Collegiate female basketball players | Basketball |
| Ribeiro et al. (2020) | RCT | 12 / 12 | 18 ± 1 | Elite international U20 female footballers | Soccer |
| Hooshmand et al. (2019) | RCT | 17 / 17 | 20–45 | Sedentary overweight women | Non-athletic population |
| Rosas et al. (2017) | RCT | 8 / 8 | 23.7 ± 2.4 | Amateur female soccer players | Soccer |
| Outlaw et al. (2016) | RCT | 7 / 8 | 21.0 ± 2.2 | Untrained collegiate females | Resistance training |
| Glenn et al. (2015/2016) (masters cyclists trial) | RCT; companion reports from the same trial | 11 / 11 | 54 ± 2 / 53 ± 1 | Female masters cyclists | Cycling |
| Kresta et al. (2014) | RCT; multi-arm trial | 8 / 7 | 21.5 ± 2.8 | Recreationally active females | Mixed training background |
| Smith et al. (2012) | RCT | 13 / 11 | 21.7 ± 2.1 | Moderately trained women | Mixed training background |
| Stout et al. (2007) | RCT | 11 / 11 | 27.4 ± 6.1 | Women | Unspecified |

**Table 1. Characteristics of the included randomized controlled trials and comparisons used in the meta-analysis (continued)**

**Panel B. Intervention, comparator, and extracted outcome details**

| **Study** | **Supplementation protocol (dose / duration)** | **Comparator** | **Supplement form** | **Co-intervention / background condition** | **Menstrual / contraceptive status** | **Blinding / side effects** | **Outcome measures** | **Specific comparison used in meta-analysis** |
| --- | --- | --- | --- | --- | --- | --- | --- | --- |
| Adamczewski et al. (2026) | 6.4 g/day; 28 days | Placebo | BA vs placebo capsules | Standardized diet before testing | NR | NR | TTE; Peak Power; Anaerobic Performance | BA+PLSB vs PLBA+PLSB; SB-containing arms excluded |
| Walter et al. (2010) | 1.5 g per dose, 4 doses/day for 21 days, then 2 doses/day for 21 days | Placebo / control | BA + dextrose powder | 6 weeks HIIT cycling | NR | NR | VO₂max; Body Fat Percentage | BA vs matched placebo/control under the same HIIT background condition |
| Gholami et al. (2022) | 6.4 g/day; 28 days | Isocaloric placebo | BA vs placebo (dextrose) | Exhaustive exercise testing | NR | NR | TTE; Peak Power; VO₂max; Body Fat Percentage | BA vs isocaloric placebo |
| Ribeiro et al. (2020) | 6.4 g/day; 21 days | Maltodextrin placebo | Sustained-release BA | 3-week standardized football-specific training camp | NR | NR | Anaerobic Performance | BA vs maltodextrin placebo under the same training-camp condition |
| Hooshmand et al. (2019) | 1.6 g/day; 42 days | Placebo | BA supplement | Usual diet and daily activity maintained | NR | NR | TTE; Body Fat Percentage | BA vs placebo |
| Rosas et al. (2017) | 4.8 g/day; 6 weeks | Placebo | Oral BA supplementation | Plyometric training | NR | Blinding success / side effects NR | Anaerobic Performance | BA vs placebo under the same plyometric-training condition |
| Outlaw et al. (2016) | 3.4 g before training sessions; 8 weeks | Placebo | BA before training sessions | 8 weeks of resistance training | NR | Blinding success / side effects NR | TTE; Peak Power; VO₂max; Body Fat Percentage | BA vs placebo under the same resistance-training condition |
| Glenn et al. (2015/2016) (masters cyclists trial) | 3.2 g/day; 28 days | Placebo | BA + dextrose | No co-intervention; usual training maintained | NR | Blinding efficacy tested; paresthesia reported in one participant | TTE; VO₂peak; Body Fat Percentage | BA vs placebo; overlapping outcomes extracted only once |
| Kresta et al. (2014) | 6.1 ± 0.7 g/day; 28 days | Placebo | BA sustained-release capsules | 4-arm trial; no additional training intervention | NR | NR | TTE; Peak Power; VO₂max; Anaerobic Performance; Body Fat Percentage | BA-only arm vs matched placebo; non-isolated active-supplement arms excluded |
| Smith et al. (2012) | 4.8 g/day; 28 days | Placebo | CarnoSyn tablets | 40-min treadmill run to induce oxidative stress | NR | NR | TTE | BA vs placebo |
| Stout et al. (2007) | 3.2 g/day for 7 days, then 6.4 g/day for 21 days; 28 days total | Placebo | CarnoSyn | No co-intervention | NR | NR | TTE | BA vs placebo |

**Notes:**

For multi-arm trials, only comparisons relevant to isolated beta-alanine supplementation were used in the pooled analyses. Glenn et al. (2015/2016) refers to two companion reports from the same 28-day beta-alanine supplementation trial in female masters cyclists; overlapping outcomes were extracted only once. For the Glenn companion reports, the sample size used in the meta-analysis was 11 / 10 for TTE and VO₂peak because one placebo-group participant withdrew due to repeated headaches and was not included in the final analyses; body fat percentage used 11 / 11.

**Abbreviations:** BA, beta-alanine; NR, not reported; PLBA, placebo beta-alanine; PLSB, placebo sodium bicarbonate; SB, sodium bicarbonate; TTE, time to exhaustion; VO₂max, maximal oxygen uptake; VO₂peak, peak oxygen uptake.

# Main manuscript Table 2. GRADE Summary of Findings

**Table 2. GRADE Summary of Findings for primary outcomes.**

| **Outcomes** | **No. of studies** | **No. of participants** | **Pooled estimate, SMD [95% CI]** | **Risk of bias** | **Inconsistency** | **Indirectness** | **Imprecision** | **Publication bias** | **Certainty (GRADE)** |
| --- | --- | --- | --- | --- | --- | --- | --- | --- | --- |
| TTE | 8 RCTs | 187 | 0.49 [0.20, 0.79] | Serious^a^ | Not serious^b^ | Not serious | Serious^d^ | Seriousᵉ | ⊕◯◯◯ Very low |
| Body fat percentage | 6 RCTs | 141 | −0.07 [−0.41, 0.26] | Serious^a^ | Not serious^b^ | Not serious | Serious^d^ | Seriousᵉ | ⊕◯◯◯ Very low |
| Peak power | 4 RCTs | 86 | 0.24 [−0.18, 0.67] | Serious^a^ | Not serious^b^ | Serious^c^ | Serious^d^ | Seriousᵉ | ⊕◯◯◯ Very low |
| Anaerobic performance | 4 RCTs | 89 | 0.11 [−0.30, 0.53] | Serious^a^ | Not serious^b^ | Serious^c^ | Serious^d^ | Seriousᵉ | ⊕◯◯◯ Very low |
| VO₂max and VO₂peak | 5 RCTs | 106 | 0.32 [−0.06, 0.71] | Serious^a^ | Not serious^b^ | Not serious | Serious^d^ | Seriousᵉ | ⊕◯◯◯ Very low |

GRADE, Grading of Recommendations Assessment, Development and Evaluation; RCT, randomized controlled trial; SMD, standardized mean difference; CI, confidence interval; TTE, time to exhaustion. Randomized controlled trials started as high-certainty evidence. Certainty was assessed separately for each outcome and was not downgraded below very low.

a. Downgraded one level for risk of bias because the contributing randomized controlled trials were judged as having some concerns in the overall RoB 2 assessment.
b. Not downgraded for inconsistency because statistical heterogeneity was minimal across pooled outcomes.
c. Downgraded one level for indirectness because the pooled outcome combined different exercise protocols, testing methods, or outcome definitions.
d. Downgraded one level for imprecision because of the limited total sample size and/or wide confidence interval around the pooled estimate.
e. Downgraded one level for publication bias because publication bias and small-study effects could not be adequately ruled out. Fewer than 10 studies contributed to each pooled outcome, which precluded reliable formal assessment of small-study effects, and the limited evidence base increased uncertainty regarding selective publication or unavailable study results.

# Supplementary Tables S1–S3. Sensitivity analysis tables

**Supplementary Sensitivity Analysis Tables**

This supplementary file reports the quantitative sensitivity analyses requested during peer review, including fixed-effect versus random-effects model comparisons, leave-one-out analyses, and REML-based random-effects sensitivity analyses.

**Supplementary Table S1. Comparison of fixed-effect and random-effects models**

| **Outcome** | **Model** | **k** | **SMD (95% CI)** | **p-value** | **Tau²** | **Q** | **Q-test p-value** | **I² (%)** |
| --- | --- | --- | --- | --- | --- | --- | --- | --- |
| TTE | Random-effects | 8 | 0.49 [0.20, 0.79] | 0.001 | 0.00 | 5.21 | 0.63 | 0 |
| TTE | Fixed-effect | 8 | 0.49 [0.20, 0.79] | 0.001 | - | 5.21 | 0.63 | 0 |
| Peak power | Random-effects | 4 | 0.24 [-0.18, 0.67] | 0.26 | 0.00 | 2.06 | 0.56 | 0 |
| Peak power | Fixed-effect | 4 | 0.24 [-0.18, 0.67] | 0.26 | - | 2.06 | 0.56 | 0 |
| VO₂max and VO₂peak | Random-effects | 5 | 0.32 [-0.06, 0.71] | 0.10 | 0.00 | 1.08 | 0.90 | 0 |
| VO₂max and VO₂peak | Fixed-effect | 5 | 0.32 [-0.06, 0.71] | 0.10 | - | 1.08 | 0.90 | 0 |
| Anaerobic performance | Random-effects | 4 | 0.11 [-0.30, 0.53] | 0.59 | 0.00 | 0.77 | 0.86 | 0 |
| Anaerobic performance | Fixed-effect | 4 | 0.11 [-0.30, 0.53] | 0.59 | - | 0.77 | 0.86 | 0 |
| Body fat percentage | Random-effects | 6 | -0.07 [-0.41, 0.26] | 0.67 | 0.00 | 5.04 | 0.41 | 1 |
| Body fat percentage | Fixed-effect | 6 | -0.07 [-0.41, 0.26] | 0.67 | - | 5.04 | 0.41 | 1 |

Note. SMD = standardized mean difference (Hedges' g); CI = confidence interval; Tau² = between-study variance; Q = Cochran's Q statistic; I² = inconsistency statistic. Tau² is not applicable to fixed-effect models and is therefore shown as '-'. Values are rounded for presentation.

**Supplementary Table S2. Leave-one-out sensitivity analyses**

| **Outcome** | **Omitted study** | **k remaining** | **SMD (95% CI)** | **p-value** | **Tau²** | **Q** | **Q-test p-value** | **I² (%)** |
| --- | --- | --- | --- | --- | --- | --- | --- | --- |
| TTE | Adamczewski et al. (2026) | 7 | 0.48 [0.16, 0.81] | 0.004 | 0.00 | 5.19 | 0.52 | 0 |
| TTE | Gholami et al. (2022) | 7 | 0.57 [0.26, 0.89] | <0.001 | 0.00 | 3.24 | 0.78 | 0 |
| TTE | Glenn et al. 2015/2016 | 7 | 0.43 [0.12, 0.75] | 0.006 | 0.00 | 3.91 | 0.69 | 0 |
| TTE | Hooshmand et al. (2019) | 7 | 0.42 [0.09, 0.74] | 0.011 | 0.00 | 4.07 | 0.67 | 0 |
| TTE | Kresta et al. (2014) | 7 | 0.47 [0.17, 0.78] | 0.002 | 0.00 | 5.02 | 0.54 | 0 |
| TTE | Outlaw et al. (2016) | 7 | 0.50 [0.19, 0.80] | 0.002 | 0.00 | 5.21 | 0.52 | 0 |
| TTE | Smith et al. (2012) | 7 | 0.50 [0.19, 0.82] | 0.002 | 0.00 | 5.17 | 0.52 | 0 |
| TTE | Stout et al. (2007) | 7 | 0.56 [0.24, 0.87] | 0.001 | 0.00 | 3.91 | 0.69 | 0 |
| Peak power | Adamczewski et al. (2026) | 3 | 0.46 [-0.10, 1.02] | 0.10 | 0.00 | 0.62 | 0.73 | 0 |
| Peak power | Gholami et al. (2022) | 3 | 0.15 [-0.35, 0.64] | 0.56 | 0.00 | 1.49 | 0.47 | 0 |
| Peak power | Kresta et al. (2014) | 3 | 0.27 [-0.21, 0.74] | 0.27 | 0.00 | 2.01 | 0.37 | 1 |
| Peak power | Outlaw et al. (2016) | 3 | 0.15 [-0.32, 0.62] | 0.52 | 0.00 | 1.18 | 0.55 | 0 |
| VO₂max and VO₂peak | Gholami et al. (2022) | 4 | 0.43 [-0.01, 0.87] | 0.054 | 0.00 | 0.07 | 1.00 | 0 |
| VO₂max and VO₂peak | Glenn et al. 2015/2016 | 4 | 0.30 [-0.13, 0.73] | 0.17 | 0.00 | 1.03 | 0.80 | 0 |
| VO₂max and VO₂peak | Kresta et al. (2014) | 4 | 0.30 [-0.11, 0.72] | 0.15 | 0.00 | 1.02 | 0.80 | 0 |
| VO₂max and VO₂peak | Outlaw et al. (2016) | 4 | 0.29 [-0.13, 0.71] | 0.17 | 0.00 | 0.89 | 0.83 | 0 |
| VO₂max and VO₂peak | Walter et al. (2010) | 4 | 0.30 [-0.16, 0.77] | 0.20 | 0.00 | 1.05 | 0.79 | 0 |
| Anaerobic performance | Adamczewski et al. (2026) | 3 | 0.17 [-0.37, 0.70] | 0.54 | 0.00 | 0.68 | 0.71 | 0 |
| Anaerobic performance | Kresta et al. (2014) | 3 | 0.05 [-0.41, 0.50] | 0.84 | 0.00 | 0.26 | 0.88 | 0 |
| Anaerobic performance | Ribeiro et al. (2020) | 3 | 0.09 [-0.40, 0.57] | 0.73 | 0.00 | 0.72 | 0.70 | 0 |
| Anaerobic performance | Rosas et al. (2017) | 3 | 0.17 [-0.29, 0.63] | 0.47 | 0.00 | 0.46 | 0.79 | 0 |
| Body fat percentage | Gholami et al. (2022) | 5 | 0.00 [-0.37, 0.37] | 0.99 | 0.01 | 4.12 | 0.39 | 3 |
| Body fat percentage | Glenn et al. 2015/2016 | 5 | 0.02 [-0.34, 0.39] | 0.91 | 0.00 | 3.34 | 0.50 | 0 |
| Body fat percentage | Hooshmand et al. (2019) | 5 | -0.07 [-0.51, 0.37] | 0.75 | 0.05 | 5.03 | 0.29 | 20 |
| Body fat percentage | Kresta et al. (2014) | 5 | -0.13 [-0.49, 0.23] | 0.48 | 0.01 | 4.11 | 0.39 | 3 |
| Body fat percentage | Outlaw et al. (2016) | 5 | -0.06 [-0.46, 0.34] | 0.77 | 0.04 | 4.94 | 0.29 | 19 |
| Body fat percentage | Walter et al. (2010) | 5 | -0.22 [-0.60, 0.16] | 0.26 | 0.00 | 2.56 | 0.63 | 0 |

Note. For each leave-one-out analysis, one study was sequentially omitted and the pooled SMD, 95% CI, p-value, Tau², Cochran's Q statistic, Q-test p-value, and I² were recalculated. SMD = standardized mean difference (Hedges' g); CI = confidence interval; TTE = time to exhaustion. Values are rounded for presentation.

**Supplementary Table S3. REML-based random-effects sensitivity analysis**

This table compares the primary DerSimonian–Laird random-effects model with a REML-based random-effects sensitivity analysis.

| **Outcome** | **Model** | **k** | **SMD (95% CI)** | **p-value** | **Tau²** | **Q** | **Q-test p-value** | **I² (%)** |
| --- | --- | --- | --- | --- | --- | --- | --- | --- |
| TTE | DL random-effects model | 8 | 0.49 [0.20, 0.79] | 0.001 | 0.00 | 5.21 | 0.63 | 0 |
| TTE | REML random-effects model | 8 | 0.49 [0.20, 0.79] | 0.001 | 0.00 | 5.21 | 0.63 | 0 |
| Peak power | DL random-effects model | 4 | 0.24 [-0.18, 0.67] | 0.26 | 0.00 | 2.06 | 0.56 | 0 |
| Peak power | REML random-effects model | 4 | 0.24 [-0.18, 0.67] | 0.26 | 0.00 | 2.06 | 0.56 | 0 |
| VO₂max and VO₂peak | DL random-effects model | 5 | 0.32 [-0.06, 0.71] | 0.10 | 0.00 | 1.08 | 0.90 | 0 |
| VO₂max and VO₂peak | REML random-effects model | 5 | 0.32 [-0.06, 0.71] | 0.10 | 0.00 | 1.08 | 0.90 | 0 |
| Anaerobic performance | DL random-effects model | 4 | 0.11 [-0.30, 0.53] | 0.59 | 0.00 | 0.77 | 0.86 | 0 |
| Anaerobic performance | REML random-effects model | 4 | 0.11 [-0.30, 0.53] | 0.59 | 0.00 | 0.77 | 0.86 | 0 |
| Body fat percentage | DL random-effects model | 6 | -0.07 [-0.41, 0.26] | 0.67 | 0.00 | 5.04 | 0.41 | 1 |
| Body fat percentage | REML random-effects model | 6 | -0.07 [-0.42, 0.27] | 0.67 | 0.01 | 5.04 | 0.41 | 1 |

Note. DL = DerSimonian–Laird; REML = restricted maximum likelihood; SMD = standardized mean difference; CI = confidence interval; Tau² = between-study variance; Q = Cochran's Q statistic; I² = inconsistency statistic. Values are rounded for presentation.

**Supplementary Table S4. Arm-level extraction decisions for multi-arm and co-intervention studies**

**Supplementary Table S4. Arm-level extraction decisions for multi-arm and co-intervention studies**

This table documents the arm-level data extracted to isolate the effect of beta-alanine while avoiding non-matched contrasts and double-counting of shared comparator groups.

| **Study** | **Trial context / background condition** | **Eligible beta-alanine arm used** | **Eligible comparator arm used** | **Arms / contrasts not extracted** | **Reason for exclusion / extraction rule** | **Sample size used (BA / comparator)** | **Outcome data extracted** | **Shared comparator reused within outcome?** |
| --- | --- | --- | --- | --- | --- | --- | --- | --- |
| Adamczewski et al. (2026) | Multi-arm supplementation trial involving beta-alanine and sodium bicarbonate; standardized diet/testing condition | BA+PLSB | PLBA+PLSB | BA+SB and PLBA+SB | SB-containing arms were not extracted because the comparison was restricted to isolated beta-alanine versus matched placebo under the same non-SB background condition. | 17 / 17 | TTE; peak power; anaerobic performance | No |
| Walter et al. (2010) | 6 weeks of HIIT cycling | Beta-alanine under the same HIIT background condition | Matched placebo/control under the same HIIT background condition | Non-matched or training-only contrasts | Only beta-alanine versus matched comparator under the same background condition was extracted. | 14 / 19 | VO₂max; body fat percentage | No |
| Gholami et al. (2022) | Exhaustive exercise testing | Beta-alanine arm | Isocaloric placebo | None | Not applicable. | 11 / 11 | TTE; peak power; VO₂max; body fat percentage | No |
| Ribeiro et al. (2020) | 3-week standardized football-specific training camp | Beta-alanine under the same training-camp condition | Maltodextrin placebo under the same training-camp condition | Non-matched or training-only contrasts | Only beta-alanine versus matched comparator under the same background condition was extracted. | 12 / 12 | Anaerobic performance | No |
| Hooshmand et al. (2019) | Usual diet and daily activity maintained | Beta-alanine arm | Placebo | None | Not applicable. | 17 / 17 | TTE; body fat percentage | No |
| Rosas et al. (2017) | Plyometric training | Beta-alanine under the same plyometric-training condition | Placebo under the same plyometric-training condition | Non-matched or training-only contrasts | Only beta-alanine versus matched comparator under the same background condition was extracted. | 8 / 8 | Anaerobic performance | No |
| Outlaw et al. (2016) | 8 weeks of resistance training | Beta-alanine under the same resistance-training condition | Placebo under the same resistance-training condition | Non-matched or training-only contrasts | Only beta-alanine versus matched comparator under the same background condition was extracted. | 7 / 8 | TTE; peak power; VO₂max; body fat percentage | No |
| Glenn et al. (2015/2016) (masters cyclists trial) | No co-intervention; usual training maintained | Beta-alanine arm | Placebo | None | The two companion reports were treated as one independent RCT; overlapping outcome data were extracted only once. | 11 / 11 | TTE; VO₂peak; body fat percentage | No |
| Kresta et al. (2014) | Multi-arm supplementation trial involving beta-alanine and creatine; no additional training intervention | BA-only | PLA | CRE-only and BAC | Creatine-containing arms were not extracted because the comparison was restricted to beta-alanine-only versus placebo. | 8 / 7 | TTE; peak power; VO₂max; anaerobic performance; body fat percentage | No |
| Smith et al. (2012) | 40-min treadmill run to induce oxidative stress | Beta-alanine arm | Placebo | None | Not applicable. | 13 / 11 | TTE | No |
| Stout et al. (2007) | No co-intervention | Beta-alanine arm | Placebo | None | Not applicable. | 11 / 11 | TTE | No |

Notes: BA, beta-alanine; BAC, beta-alanine plus creatine; CRE, creatine; HIIT, high-intensity interval training; PLA, placebo; PLBA, placebo as beta-alanine; PLSB, placebo as sodium bicarbonate; RCT, randomized controlled trial; SB, sodium bicarbonate; TTE, time to exhaustion; VO₂max, maximal oxygen uptake; VO₂peak, peak oxygen uptake. Eligible arms were defined as beta-alanine-only versus matched placebo/control under the same background condition. Active-supplement combination arms and non-matched contrasts were not extracted for the primary pooled analyses. *For the Glenn companion reports, 11 / 11 reflects the original randomized BA and placebo groups. The sample size used in the meta-analysis was 11 / 10 for TTE and VO₂peak because one placebo-group participant withdrew due to repeated headaches and was not included in the final analyses; body fat percentage used 11 / 11.

# Supplementary Table S5. Report-to-study mapping table

**Supplementary Table S5. Report-to-study mapping table**

This table maps each included report to the independent randomized controlled trial represented in the review and documents decisions used to avoid participant double-counting in the meta-analysis.

| **Report** | **Independent study represented** | **Status in review** | **Relationship to other reports / sample overlap** | **Outcomes extracted for meta-analysis** | **Participant overlap and double-counting decision** |
| --- | --- | --- | --- | --- | --- |
| Adamczewski et al. (2026) | Adamczewski (2026) | Included report; independent RCT | No participant overlap with other included reports was identified. | TTE; peak power; anaerobic performance | Counted once as an independent sample in each relevant pooled outcome. |
| Walter et al. (2010) | Walter (2010) | Included report; independent RCT | No participant overlap with other included reports was identified. | VO₂max; body fat percentage | Counted once as an independent sample in each relevant pooled outcome. |
| Gholami et al. (2022) | Gholami (2022) | Included report; independent RCT | No participant overlap with other included reports was identified. | TTE; peak power; VO₂max; body fat percentage | Counted once as an independent sample in each relevant pooled outcome. |
| Ribeiro et al. (2020) | Ribeiro (2020) | Included report; independent RCT | No participant overlap with other included reports was identified. | Anaerobic performance | Counted once as an independent sample in the relevant pooled outcome. |
| Hooshmand et al. (2019) | Hooshmand (2019) | Included report; independent RCT | No participant overlap with other included reports was identified. | TTE; body fat percentage | Counted once as an independent sample in each relevant pooled outcome. |
| Rosas et al. (2017) | Rosas (2017) | Included report; independent RCT | No participant overlap with other included reports was identified. | Anaerobic performance | Counted once as an independent sample in the relevant pooled outcome. |
| Outlaw et al. (2016) | Outlaw (2016) | Included report; independent RCT | No participant overlap with other included reports was identified. | TTE; peak power; VO₂max; body fat percentage | Counted once as an independent sample in each relevant pooled outcome. |
| Glenn et al. (2015) | Glenn masters cyclists trial | Included companion report | Same 28-day beta-alanine trial as Glenn et al. (2016); participants overlap with the companion report. | TTE; VO₂peak | Same participant sample as Glenn et al. (2016). This report contributed TTE and VO₂peak data. It was treated as part of the same independent RCT, and no outcome data were double-counted. |
| Glenn et al. (2016) | Glenn masters cyclists trial | Included companion report | Same 28-day beta-alanine trial as Glenn et al. (2015); participants overlap with the companion report. | Body fat percentage | Same participant sample as Glenn et al. (2015). This report contributed body fat percentage data. It was treated as part of the same independent RCT, and no outcome data were double-counted. |
| Kresta et al. (2014) | Kresta (2014) | Included report; independent RCT | No participant overlap with other included reports was identified. | TTE; peak power; VO₂max; anaerobic performance; body fat percentage | Counted once as an independent sample in each relevant pooled outcome; no shared comparator group was double-counted. |
| Smith et al. (2012) | Smith (2012) | Included report; independent RCT | No participant overlap with other included reports was identified. | TTE | Counted once as an independent sample in the relevant pooled outcome. |
| Stout et al. (2007) | Stout (2007) | Included report; independent RCT | No participant overlap with other included reports was identified. | TTE | Counted once as an independent sample in the relevant pooled outcome. |

**Notes:** The review included 12 reports from 11 independent RCTs. Glenn et al. (2015) and Glenn et al. (2016) originated from the same 28-day beta-alanine supplementation trial and were treated as one independent study; overlapping outcome data were extracted only once. BA, beta-alanine; RCT, randomized controlled trial; TTE, time to exhaustion; VO₂max, maximal oxygen uptake; VO₂peak, peak oxygen uptake.

# Supplementary Table S6. Outcome-level RoB 2 assessment and blinding-related information

**Supplementary Table S6. Outcome-level RoB 2 assessment and blinding-related information**

This table presents the outcome-level RoB 2 judgments for each study-outcome contribution included in the meta-analysis. Where domain judgments did not differ across outcomes within a study, the same D1-D5 judgments are repeated across the relevant outcome rows.

| **Outcome domain** | **Study** | **D1 Randomization** | **D2 Deviations** | **D3 Missing data** | **D4 Outcome measurement** | **D5 Selective reporting** | **Overall RoB 2** | **Blinding / side effects** | **Placebo matching / supplement form** | **Assessor blinding** |
| --- | --- | --- | --- | --- | --- | --- | --- | --- | --- | --- |
| TTE | Adamczewski et al. (2026) | Low | Low | Some concerns | Some concerns | Low | Some concerns | NR | BA/placebo capsules; SB arms excluded | NR |
| TTE | Gholami et al. (2022) | Some concerns | Low | Low | Some concerns | Some concerns | Some concerns | NR | BA vs dextrose placebo | NR |
| TTE | Glenn et al. (2015/2016) | Some concerns | Low | Low | Some concerns | Some concerns | Some concerns | Blinding efficacy tested; paresthesia reported in one participant | BA + dextrose vs placebo | NR |
| TTE | Hooshmand et al. (2019) | Low | Low | Some concerns | Some concerns | Low | Some concerns | NR | BA supplement vs placebo | NR |
| TTE | Kresta et al. (2014) | Some concerns | Some concerns | Some concerns | Some concerns | Low | Some concerns | NR | BA sustained-release capsules vs PLA; creatine arms excluded | NR |
| TTE | Outlaw et al. (2016) | Some concerns | Low | Low | Some concerns | Some concerns | Some concerns | Blinding success/side effects NR | BA vs placebo | NR |
| TTE | Smith et al. (2012) | Low | Low | Low | Some concerns | Some concerns | Some concerns | NR | CarnoSyn tablets vs placebo | NR |
| TTE | Stout et al. (2007) | Some concerns | Low | Low | Some concerns | Some concerns | Some concerns | NR | CarnoSyn vs placebo | NR |
| Peak power | Adamczewski et al. (2026) | Low | Low | Some concerns | Some concerns | Low | Some concerns | NR | BA/placebo capsules; SB arms excluded | NR |
| Peak power | Gholami et al. (2022) | Some concerns | Low | Low | Some concerns | Some concerns | Some concerns | NR | BA vs dextrose placebo | NR |
| Peak power | Kresta et al. (2014) | Some concerns | Some concerns | Some concerns | Some concerns | Low | Some concerns | NR | BA sustained-release capsules vs PLA; creatine arms excluded | NR |
| Peak power | Outlaw et al. (2016) | Some concerns | Low | Low | Some concerns | Some concerns | Some concerns | Blinding success/side effects NR | BA vs placebo | NR |
| VO₂max and VO₂peak | Gholami et al. (2022) | Some concerns | Low | Low | Some concerns | Some concerns | Some concerns | NR | BA vs dextrose placebo | NR |
| VO₂max and VO₂peak | Glenn et al. (2015/2016) | Some concerns | Low | Low | Some concerns | Some concerns | Some concerns | Blinding efficacy tested; paresthesia reported in one participant | BA + dextrose vs placebo | NR |
| VO₂max and VO₂peak | Kresta et al. (2014) | Some concerns | Some concerns | Some concerns | Some concerns | Low | Some concerns | NR | BA sustained-release capsules vs PLA; creatine arms excluded | NR |
| VO₂max and VO₂peak | Outlaw et al. (2016) | Some concerns | Low | Low | Some concerns | Some concerns | Some concerns | Blinding success/side effects NR | BA vs placebo | NR |
| VO₂max and VO₂peak | Walter et al. (2010) | Some concerns | Some concerns | Low | Some concerns | Some concerns | Some concerns | NR | BA + dextrose vs placebo/control | NR |
| Anaerobic performance | Adamczewski et al. (2026) | Low | Low | Some concerns | Some concerns | Low | Some concerns | NR | BA/placebo capsules; SB arms excluded | NR |
| Anaerobic performance | Kresta et al. (2014) | Some concerns | Some concerns | Some concerns | Some concerns | Low | Some concerns | NR | BA sustained-release capsules vs PLA; creatine arms excluded | NR |
| Anaerobic performance | Ribeiro et al. (2020) | Low | Low | Some concerns | Some concerns | Some concerns | Some concerns | NR | Sustained-release BA vs maltodextrin placebo | NR |
| Anaerobic performance | Rosas et al. (2017) | Low | Some concerns | Some concerns | Some concerns | Some concerns | Some concerns | Blinding success/side effects NR | Oral BA vs placebo | NR |
| Body fat percentage | Gholami et al. (2022) | Some concerns | Low | Low | Some concerns | Some concerns | Some concerns | NR | BA vs dextrose placebo | NR |
| Body fat percentage | Glenn et al. (2015/2016) | Some concerns | Low | Low | Some concerns | Some concerns | Some concerns | Blinding efficacy tested; paresthesia reported in one participant | BA + dextrose vs placebo | NR |
| Body fat percentage | Hooshmand et al. (2019) | Low | Low | Some concerns | Some concerns | Low | Some concerns | NR | BA supplement vs placebo | NR |
| Body fat percentage | Kresta et al. (2014) | Some concerns | Some concerns | Some concerns | Some concerns | Low | Some concerns | NR | BA sustained-release capsules vs PLA; creatine arms excluded | NR |
| Body fat percentage | Outlaw et al. (2016) | Some concerns | Low | Low | Some concerns | Some concerns | Some concerns | Blinding success/side effects NR | BA vs placebo | NR |
| Body fat percentage | Walter et al. (2010) | Some concerns | Some concerns | Low | Some concerns | Some concerns | Some concerns | NR | BA + dextrose vs placebo/control | NR |

**Notes:** D1, bias arising from the randomization process; D2, bias due to deviations from intended interventions; D3, bias due to missing outcome data; D4, bias in measurement of the outcome; D5, bias in selection of the reported result; BA, beta-alanine; NR, not reported; PLA, placebo; PLBA, placebo beta-alanine; PLSB, placebo sodium bicarbonate; RoB 2, Cochrane Risk of Bias 2 tool; SB, sodium bicarbonate; TTE, time to exhaustion; VO2max, maximal oxygen uptake; VO2peak, peak oxygen uptake. Judgments are reported at the outcome level for the outcomes included in the pooled analyses. D4 judgments considered expectancy effects, blinding success, side effects, placebo matching, supplement form, and assessor blinding where reported. No study-outcome contribution was judged as high risk of bias overall.

**Supplementary Table S7. Database-specific search strategies**

**Table S7. Search Strategy.**

| **Search Strategy** | |
| --- | --- |
| PubMed | ("beta-Alanine"[Mesh] OR "beta-alanine"[Title/Abstract] OR "beta alanine"[Title/Abstract] OR "β-alanine"[Title/Abstract] OR carnosine[Title/Abstract]) AND ("Female"[Mesh] OR women[Title/Abstract] OR woman[Title/Abstract] OR female[Title/Abstract] OR females[Title/Abstract]) AND ("Athletic Performance"[Mesh] OR "Exercise"[Mesh] OR "Sports"[Mesh] OR "exercise performance"[Title/Abstract] OR sport*[Title/Abstract] OR athletic*[Title/Abstract] OR "time to exhaustion"[Title/Abstract] OR TTE[Title/Abstract] OR VO₂max[Title/Abstract] OR VO₂peak[Title/Abstract] OR "peak power"[Title/Abstract] OR "anaerobic performance"[Title/Abstract] OR "body fat percentage"[Title/Abstract] OR "body composition"[Title/Abstract]) |
| Web of Science | #1 ALL=("beta-alanine" OR "beta alanine" OR "β-alanine" OR carnosine)  #2 ALL=(women OR woman OR female OR females)  #3 ALL=(exercise OR sport* OR athletic* OR performance OR "time to exhaustion" OR TTE OR VO₂max OR VO₂peak OR "peak power" OR "anaerobic performance" OR "body fat percentage" OR "body composition")  #4 #1 AND #2 AND #3 |
| Scopus | TITLE-ABS-KEY(("beta-alanine" OR "beta alanine" OR "β-alanine" OR carnosine) AND (women OR woman OR female OR females) AND ("athletic performance" OR exercise OR sport* OR athletic* OR performance OR "exercise performance" OR "time to exhaustion" OR TTE OR VO₂max OR VO₂peak OR "peak power" OR "anaerobic performance" OR "body fat percentage" OR "body composition")) |
| Cochrane Library | #1 ("beta-alanine" OR "beta alanine" OR "β-alanine" OR carnosine):ti,ab,kw  #2 (women OR woman OR female OR females):ti,ab,kw  #3 ("athletic performance" OR exercise OR sport* OR athletic* OR performance OR "time to exhaustion" OR TTE OR VO₂max OR VO₂peak OR "peak power" OR "anaerobic performance" OR "body fat percentage" OR "body composition"):ti,ab,kw  #4 #1 AND #2 AND #3 |
| Embase | ('beta-alanine':ti,ab,kw OR 'beta alanine':ti,ab,kw OR 'β-alanine':ti,ab,kw OR carnosine:ti,ab,kw) AND (women:ti,ab,kw OR woman:ti,ab,kw OR female:ti,ab,kw OR females:ti,ab,kw) AND (exercise:ti,ab,kw OR sport*:ti,ab,kw OR athletic*:ti,ab,kw OR performance:ti,ab,kw OR 'time to exhaustion':ti,ab,kw OR TTE:ti,ab,kw OR VO₂max:ti,ab,kw OR VO₂peak:ti,ab,kw OR 'peak power':ti,ab,kw OR 'anaerobic performance':ti,ab,kw OR 'body fat percentage':ti,ab,kw OR 'body composition':ti,ab,kw) |

Search strategies were adapted to the syntax and indexing system of each database. Searches were conducted on April 30, 2026, from database inception to April 30, 2026. No publication-year restriction was applied. Only English-language articles were considered during eligibility screening.

**Supplementary Figure S1 description:** Forest plots showing the standardized mean differences (Hedges’ g) and 95% confidence intervals for five outcomes: time to exhaustion (TTE), peak power, VO₂max and VO₂peak, anaerobic performance, and body fat percentage. Each plot displays individual study-level effects, pooled estimates, heterogeneity statistics, study weights, and prediction intervals.

**
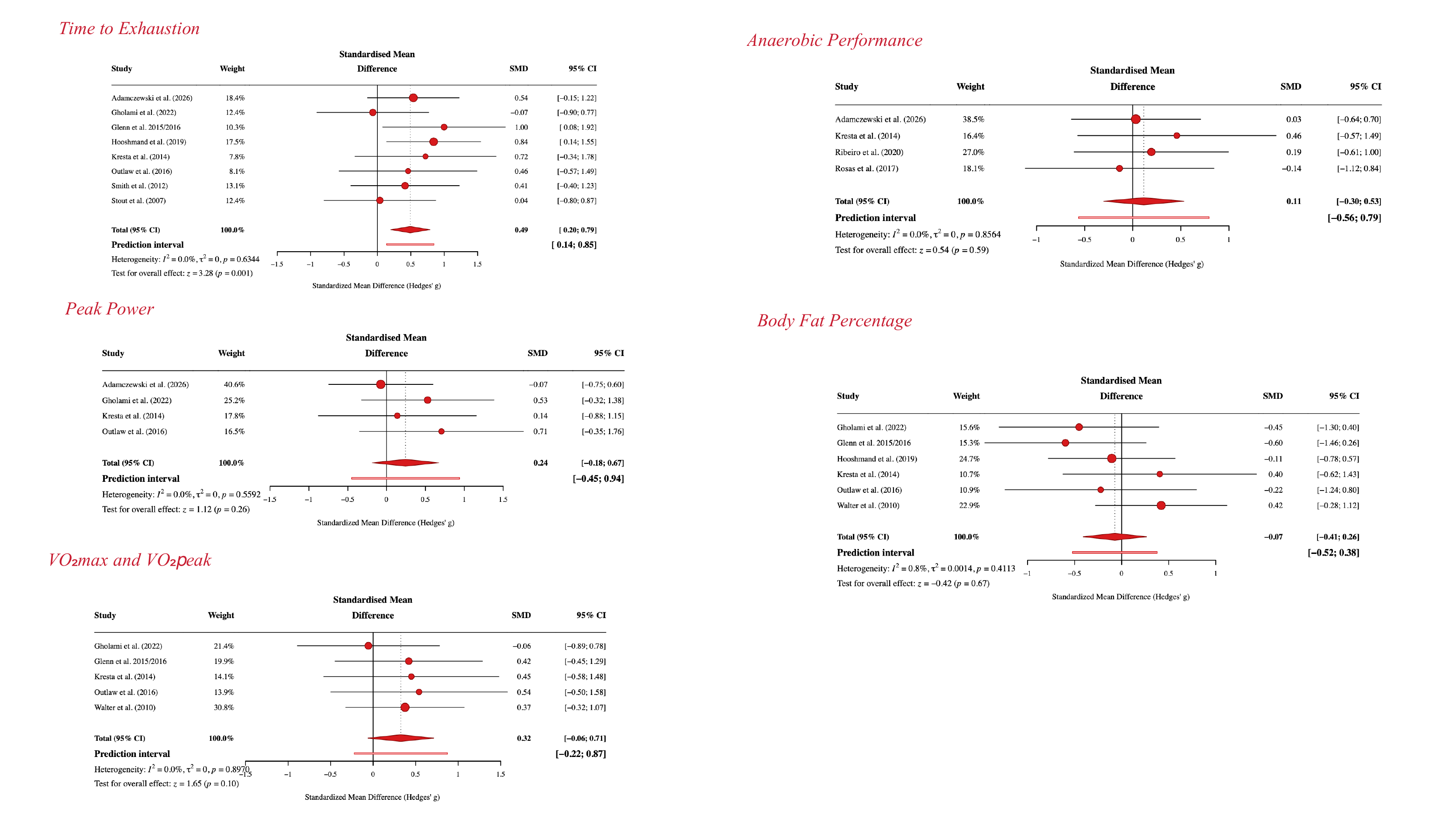
**
